# Malaria in Africa: Vector Species' Niche Models and Relative Risk Maps

**DOI:** 10.1371/journal.pone.0000824

**Published:** 2007-09-05

**Authors:** Alexander Moffett, Nancy Shackelford, Sahotra Sarkar

**Affiliations:** Section of Integrative Biology, University of Texas at Austin, Austin, Texas, United States of America; National AIDS Research Institute, India

## Abstract

A central theoretical goal of epidemiology is the construction of spatial models of disease prevalence and risk, including maps for the potential spread of infectious disease. We provide three continent-wide maps representing the relative risk of malaria in Africa based on ecological niche models of vector species and risk analysis at a spatial resolution of 1 arc-minute (9 185 275 cells of approximately 4 sq km). Using a maximum entropy method we construct niche models for 10 malaria vector species based on species occurrence records since 1980, 19 climatic variables, altitude, and land cover data (in 14 classes). For seven vectors (*Anopheles coustani*, *A*. *funestus*, *A*. *melas*, *A*. *merus*, *A. moucheti*, *A*. *nili*, and *A*. *paludis*) these are the first published niche models. We predict that Central Africa has poor habitat for both *A*. *arabiensis* and *A*. *gambiae*, and that *A*. *quadriannulatus* and *A*. *arabiensis* have restricted habitats in Southern Africa as claimed by field experts in criticism of previous models. The results of the niche models are incorporated into three relative risk models which assume different ecological interactions between vector species. The “additive” model assumes no interaction; the “minimax” model assumes maximum relative risk due to any vector in a cell; and the “competitive exclusion” model assumes the relative risk that arises from the most suitable vector for a cell. All models include variable anthrophilicity of vectors and spatial variation in human population density. Relative risk maps are produced from these models. All models predict that human population density is the critical factor determining malaria risk. Our method of constructing relative risk maps is equally general. We discuss the limits of the relative risk maps reported here, and the additional data that are required for their improvement. The protocol developed here can be used for any other vector-borne disease.

## Introduction

A central theoretical goal of epidemiology is the construction of spatial models of disease prevalence and risk, including maps for the potential spread of infectious disease [1], [2]. In particular, Snow *et al*. [3] have emphasized the need for risk maps for malaria in Africa which accounts for an estimated 85% of the 1 million annual deaths due to this disease [4]. Risk maps can be used to identify appropriate strategies of response to disease outbreaks including targeted vaccination [5] and vector, reservoir, or agent control [6]. Risk maps have been constructed using a variety of techniques including reports of disease cases [7] and distributions of disease agents, reservoirs, or vectors, based on surveys and expert opinion [8]. In recent years, these methods have been extended to use ecological models to predict the potential spatial spread of disease [9]. The use of ecological niche models for quantitative prediction of geographical distributions of agent, reservoir, and vector species has been advocated to augment traditional mapping methods such as splining and kriging [9]. Here we report a systematic attempt to construct niche models for all vectors of malaria in Africa for which data are available.

Niche models predict the “fundamental niche” of a species which identifies the region in ecological space that the species would occupy were its movement unrestricted [9], [10]. Within biodiversity studies, in which the concern is typically with the conservation of the extant habitat of a species, this fundamental niche (representing a potential distribution) is typically restricted to a realized geographical niche by using additional information such as confirmed occurrence records for a species within each accepted contiguous piece of modelled suitable habitat [10]. Such a choice is conservative in the sense that a species may occur outside its predicted realized niche. In typical contexts of biodiversity conservation planning such conservatism is appropriate; it ensures that areas selected for conservation management are maximally likely to contain the species predicted for them. However, in epidemiological contexts, especially if the interest is in identifying risk, the geographical extent of the fundamental niche is more relevant as this range defines the areas to which agent, reservoir, and vector species may potentially spread because of ecological suitability. (This means that, in epidemiological contexts, the fundamental niche or potential distribution should not be clipped to a smaller realized niche in most cases.) In this analysis, fundamental niches as predicted by niche models are used as the basis for the construction of relative risk maps.

We construct niche models for 10 *Anopheles* species recognized as vectors of malaria in Africa using a maximum entropy method based on known species' occurrences and environmental layers. These niche models predict geographic distributions of the species. For seven of these species the results presented here appear to be the first niche models reported in the literature. We used the Maxent software package [11] for the maximum entropy modelling. Within biodiversity studies, in which niche modelling is a standard technique [12]–[16] this maximum entropy method has emerged as one of the three most reliable techniques for predicting species' distributions [11], [17]. The other two most reliable methods are genetic algorithms (GARP [18]) and regression trees [19]. The advantage of Maxent over GARP is that it is much faster and allows for the simultaneous modelling of an indefinite number of species. The advantage of maximum entropy methods over both genetic algorithms and regression methods is that Maxent predicts relative probabilities of occurrence rather than the simple presence or absence of a species. This permits a finer (more nuanced) risk assessment than what can be achieved from presence-absence predictions alone. Our analysis appears to be the first use of Maxent in an epidemiological context.

Using the niche models, spatial information on human population densities, and the human blood index (HBI) values of *Anopheles* species for which these values were available, we construct preliminary relative risk maps for malaria in Africa. These maps report the relative risk of malaria occurrence at different geographical locations. We discuss in detail what data are necessary to make such maps more accurate and how our methods can be generalized to other vector-borne diseases.

There have been five recent vector-based attempts to construct risk maps for malaria. Kiszewski *et al*. [8] produced a global risk map based on the stability of malaria parasite transmission. They divided the world into 260 malarious regions and then identified the dominant vector in each region. The dominant vector was defined as the most abundant *Anopheles* species in the region that was a malaria vector, contained sporozoites frequently, and fed predominantly on humans. The stability index of a region was defined using the human feeding rate, daily survival rate, and length of the extrinsic incubation period of the dominant vector. The effects of temperature and precipitation on these parameters were quantified and environmental data were used to produce a worldwide projection of malaria transmission.

Lindsay *et al.* [20] used nonlinear regression to relate known occurrences of *A*. *arabiensis* and *A*. *gambiae* to environmental parameters. The regression was used to predict the relative proportion of the two species throughout Africa. Kuhn *et al*. [21] based their map on a database of occurrence records of six *Anopheles* species in Europe. Statistical analyses correlated these occurrence points with environmental parameters. The correlations were used to predict the distribution of the *Anopheles* species across Europe. Rogers *et al*. [22] used a maximum likelihood analysis to identify the optimal environmental parameters to predict the occurrence of five *Anopheles* species in Africa. Levine *et al*. [23], [24] used niche models to map malaria risk in Africa, Central America, and the United States. Using species' occurrence and environmental data in a genetic algorithm (in the GARP software package), they predicted the distribution of three *Anopheles* species in Africa and five species in the United States.

In addition to these vector-based approaches, risk maps have also been constructed by mapping the distribution of the malaria parasite. Kleinschmidt *et al*. [25], [26] used regression analysis to determine the relationship between the malaria parasite prevalence of an area and environmental variables. The regression model was refined using kriging for spatial interpolation thus producing a map of malaria prevalence. Diggle *et al.* [27] used a generalized linear mixed model to determine the relationship between malaria presence in children and their age and bed net use along with available medical services and land cover. Gemperli *et al*. [7] developed a Bayesian model to calculate parasite transmission intensity on the basis of malaria survey information and land cover, temperature, and rainfall data.

We map malaria risk using distributions of malaria vectors rather than malaria parasites. The underlying assumption is that malaria vector abundance is an adequate surrogate for malaria risk without explicit inclusion of parasite abundance. However, even if this assumption is invalid, risk maps based on vector distributions are still useful to augment risk analyses based only on parasite distributions. (In future work, we plan to construct risk maps using more sophisticated models of transmission that include interactions between parasites, vectors, and variable human susceptibility [28]–[30].)

This analysis differs from and extends previous efforts in five ways. First, our niche models are constructed using a maximum entropy method. Second, we model a larger number of species than previously attempted. Third, we include human population distribution data in the risk analysis and associated relative risk models. Fourth, we perform a sensitivity analysis to evaluate the robustness of our results. Fifth, we show how additional data can lead to a more sophisticated risk analysis for malaria and other vector-borne diseases.

## Methods

### Data

A 1 arc-minute (0.01666°×0.01666° longitude×latitude, approximately 4 km^2^ at the equator) grid was used to divide Africa into 9 185 275 cells. Twenty environmental layers were obtained from the WorldClim database [31]. Each layer was available at a resolution of 30 arc-seconds (0.008333°×0.008333°) and was resampled at a resolution of 1 arc-minute. These layers are listed in Table 1. (All spatial data manipulation used ArcMap GIS [32].)

**Table 1:** Environmental Parameters Used in Niche Modelling

Parameter
Annual Mean Temperature
Mean Diurnal Range
Isothermality
Temperature Seasonality
Maximum Temperature of Warmest Month
Minimum Temperature of Coldest Month
Temperature Annual Range
Mean Temperature of Wettest Quarter
Mean Temperature of Driest Quarter
Mean Temperature of Warmest Quarter
Mean Temperature of Coldest Quarter
Annual Precipitation
Precipitation of Wettest Month
Precipitation of Driest Month
Precipitation Seasonality
Precipitation of Wettest Quarter
Precipitation of Driest Quarter
Precipitation of Warmest Quarter
Precipitation of Coldest Quarter
Altitude
Land Cover

Land cover data were obtained from the Global Land Cover Facility [33]. AVHRR satellite data acquired between 1981 and 1994 were used to derive 14 classes of land cover: water; evergreen needleleaf forest; evergreen broadleaf forest; deciduous needleleaf forest; deciduous broadleaf forest; mixed forest; woodland; wooded grassland; closed shrubland; open shrubland; grassland; cropland; bare ground; urban and built. The data were initially available at a resolution of 1 km^2^ and resampled at a resolution of 1 arc-minute.

A review of the global distribution of malaria vectors [34] was used to identify those African *Anopheles* species that are capable of transmitting malaria. The 29 species used for this analysis are listed in Table 2. An extensive literature search was performed to obtain records of vector occurrences. Besides using the Mapping Malaria Risk in Africa (MARA) database [3] both PubMed and Google Scholar were searched using “Africa” and “distribution” in conjunction with the names of each of the *Anopheles* species listed in Table 2. References from those papers so identified were also searched. This resulted in a data set of 3 342 records of 22 malaria vectors, with 2634 of the records drawn from the MARA database.

**Table 2:** Occurrence Data used in Niche Modelling

Species	Records from the MARA Database	References	Additional Records	References
*A. arabiensis*	129	[53]–[100]	292	[101]–[126]
*A. aruni*	0		0	
*A. atroparvus*	0		0	
*A. brunnipes*	0		2	[127], [128]
*A. coustani*	0		22	[101], [111], [114], [115], [118], [120], [125], [128]–[138]
*A. d'thali*	0		0	
*A. flavicosta*	0		3	[128], [131], [139]
*A. funestus*	0		64	[101], [102], [106], [108], [111], [112], [114], [115], [118], [120], [126], [127], [129], [131]–[134], [136], [137], [138], [140]–[152]
*A. gambiae*	139	[54], [57], [59], [61], [63]–[67], [70], [73], [75], [77]–[80], [84]–[92], [97], [99], [127], [153]–[163]	364	[103], [104], [106], [108], [112], [113], [116], [117], [121], [122], [124], [132], [135]–[138], [144], [145], [147], [151], [164]–[172]
*A. hancocki*	0		9	[127]–[129], [131], [133], [137], [139], [141]
*A. hagreavesi*	0		0	
*A. hispaniola*	0		0	
*A. labranchiae*	0		0	
*A. marshallii*	0		12	[101], [120], [138], [173]
*A. melas*	29	[54], [64], [65], [86], [89], [97], [153], [157]	34	[103], [108], [124], [126]
*A. merus*	33	[59], [72], [73], [78]–[80], [83], [88], [99], [174], [175]	39	[107], [113]
*A. moucheti*	0		15	[101], [115], [129], [133], [137], [138], [141], [151], [166], [176]
*A. multicolor*	0		2	[176]
*A. nili*	0		16	[120], [128], [129], [131], [133], [138], [141], [151], [177], [178]
*A. paludis*	0		9	[127], [129], [133], [137], [138], [151]
*A. pharoensis*	0		19	[101], [109], [111], [112], [115], [118], [120], [125], [128], [131], [132], [134], [139], [145], [179]
*A. pretoriensis*	0		4	[115], [128], [134], [139]
*A. quadriannulatus*	33	[59], [60], [72], [81], [88], [93], [99]	36	[107], [110], [180]
*A. rhodesiensis*	0		0	
*A. rufipes*	0		13	[106], [111], [112], [115], [118], [126], [128], [131], [133], [134], [139]
*A. sergentii*	0		2	[179]
*A. squamosus*	0		6	[128], [131]–[133], [135], [139]
*A. wellcomei*	0		1	[141]
*A. ziemanni*	0		13	[118], [128], [129], [131]–[133], [138], [139], [141], [145]

Included in column (i) are each of the 29 *Anopheles* species responsible for the spread of malaria in Africa. Column (ii) contains the number of records drawn from the MARA database for each species. Column (iii) contains the references from which the MARA data were obtained. Column (iv) contains the number of records drawn from sources not included in the MARA database. Column (v) contains the references from which these additional records were obtained.

Each record was georeferenced to the nearest arc minute and assigned to a corresponding cell. For those cells containing more than one record of a given species only the most recent record was kept. So as to increase the likelihood that current values of the selected environmental parameters represent the environment as it existed when the records were obtained, only those records reporting observations made after 1980 were included. The resulting data set consisted of 977 records of 22 *Anopheles* species with 367 of these records drawn from the MARA database. Table 2 provides a summary of these records.

**Figure 1:**
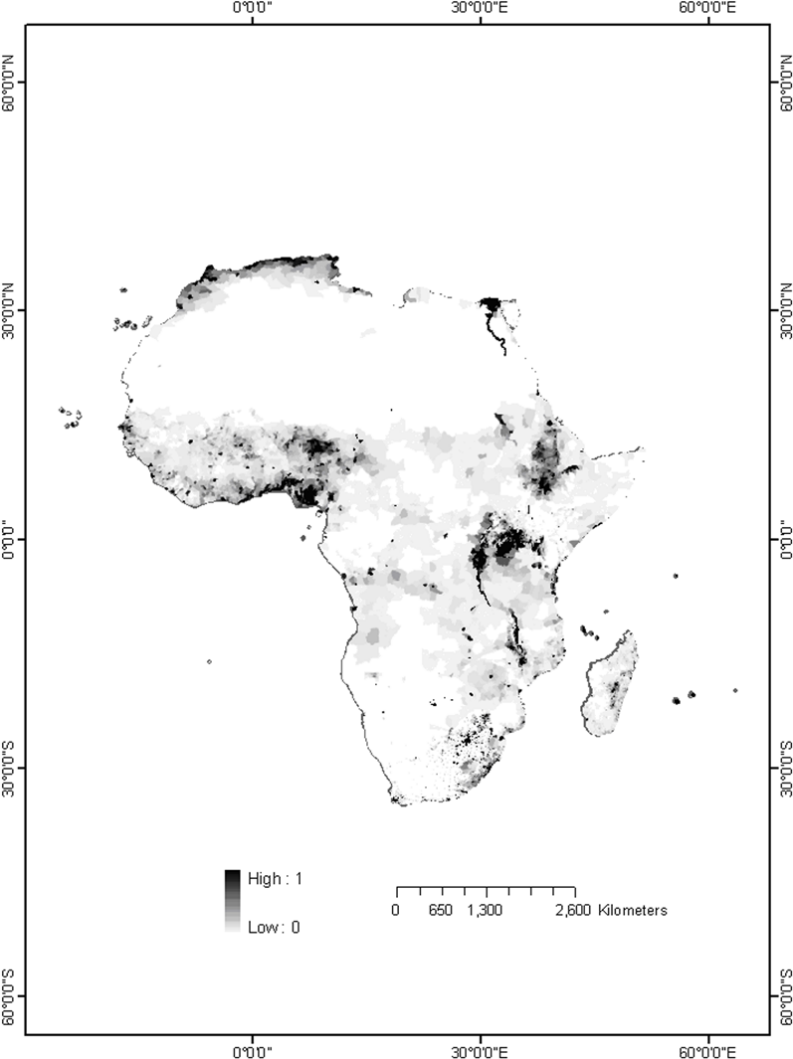
Population density in Africa. The population densities have been normalized so as to range over the unit interval.

Human population density data for the year 2000 were obtained from the Gridded Population of the World database [35]. These data were provided at a resolution of 2.5 arc-minutes (0.041666°×0.041666°) and resampled at a resolution of 1 arc-minute. Figure 1 shows a map of normalized population densities.

HBI values were obtained from the literature. Both PubMed and Google Scholar were searched using the terms “*Anopheles*” and “human blood index” with each of the species names. References from those papers so identified were also searched. Of the 10 species for which we were able to construct reliable niche models (see below), HBI values were available for nine species, with no value available for *A. paludis*.

### Niche Models

Previous niche models of *Anopheles* species have used genetic algorithms, as implemented in GARP [23], [24]. In this analysis we use instead a maximum entropy technique implemented in the Maxent software package [11]. Maxent was used instead of GARP because Maxent provides relative probabilities of presence rather than only presence-absence output and because it has, in some recent studies, been shown to outperform GARP with respect to its predictive success [11], [17]. However, there are some preliminary data suggesting that GARP may perform better than Maxent at extrapolating from occurrence data (A. Townsend Peterson, personal communication). This means that models constructed using Maxent may be excessively conservative thus predicting false absences more often than GARP. If this is true then while the areas of high relative risk identified in our maps are probably reliable, those with low relative risk should not be entirely discounted. Regression tree methods (for instance those implemented in the RandomForest software package [19]) have so far not been used in an epidemiological context.

Given a set of records of species occurrences and values of selected environmental variables defined over a chosen geographical region, Maxent predicts the distribution of species in that space by finding the distribution of maximum entropy subject to the constraint that the expectation of the distribution of each species matches its observed average over the sample locations [11]. The distribution thus produced is a relative probability distribution for each species over all cells. If each relative probability in a cell is divided by the maximum such probability across the landscape, then the result is a normalized relative probability distribution which assumes that the species is certainly present in the cell in which it had its highest predicted relative probability. This analysis used this normalization.

Computer memory limitations prevented Maxent runs using as input all 9 185 175 cells simultaneously. Instead, Maxent was run using 100 sets of 10 000 cells drawn randomly from the complete set. Sets of 10 000 cells were used because Maxent performance does not significantly improve when more than 10 000 cells are used (S. Phillips, personal communication), while 100 sets were selected to sample widely from the complete set of cells. The 100 different niche models produced for each species were averaged and subsequently normalized as described above. As we were concerned with the geographical range of the fundamental niche of the vector species, these output maps were not further refined.

The accuracy of the niche models thus produced was evaluated by constructing the models using only 75% of the records with the remaining 25% set aside for testing. The accuracy of each model was then determined by performing both a threshold-dependent binomial test of omission and a threshold-independent receiver operating characteristic analysis with those cells set aside during model development [11].

In the threshold-dependent binomial test of omission a threshold of 0.10 was used to classify each vector as either present or absent in each cell, with a vector present in a given cell if the niche model assigned it a value greater than 0.10 in that cell and absent otherwise. This threshold transformed the continuous data produced by the niche models into binary data to allow a binomial test to be performed. For each vector the number of cells in which the vector was predicted to be present was compared to the number of cells known to contain the vector. A one tailed binomial test was used to determine whether the niche model outperformed a random model predicting the vector to be present in the same number of cells [11]. Maxent provides test statistics for binomial tests for 10 different threshold values. The value of 0.10 used in this analysis was arbitrarily selected from this set of possible threshold values.

In the threshold-independent receiver operating characteristic analysis, the sensitivity and specificity of the niche models were calculated at all possible thresholds. The sensitivity of a model at a threshold was defined as the percentage of species occurrences that were correctly predicted by the model at the threshold, while the specificity of the model at a threshold was defined as the percentage of correctly predicted species absences at the threshold [11]. By calculating the sensitivity and specificity of a model at all possible thresholds a receiver operating characteristic curve was produced with sensitivity plotted on the *y*-axis and (1–specificity) plotted on the *x*-axis. The area under the curve (AUC) of the resulting plot provides a measure of model performance independent of the choice of any particular threshold. An optimal model, one that predicted each occurrence of a species and for which each prediction was accurate, would have an AUC of 1.0 while a model that predicted species occurrences at random would have an AUC of 0.5.

These two tests were used to restrict attention to those models that performed significantly better than random. Only those niche models possessing both a *p* value less than 0.05 for the binomial test of omission and an AUC greater than 0.75 were used in this analysis. The same protocol for model retention has previously been used by Pawar *et al*. [16].

### Risk Models

Three different relative risk models were constructed in which a value between 0 and 1 was assigned to each cell representing the relative risk of malaria posed to the human population residing within it. These models only used the nine species for which an HBI value was available. The models only incorporate the risk from ecological and demographic factors and ignore the modulation of risk through human intervention such as measures to control the spread of parasites or vectors. They also assume that parasites are present at sufficient densities to be capable of spreading whenever vectors are present. These assumptions are generally appropriate for Africa given the continued prevalence of malaria within it. However they would not be appropriate for regions such as northern Australia from which malaria parasites have been eliminated though malaria vectors remain

Let a*_lik_* be the relative abundance of the *l*-th vector for the *i*-th parasite in cell *k* (relative to other cells in the landscape). Let *p_k_* be the human population of cell *k*. Let h*_lik_* be the HBI of the *l*-th vector for the *i*-th parasite in cell *k*. Let e*_lik_* be the transmission efficiency of the *l*-th vector for the *i*-th parasite in cell *k*, measured by the relative likelihood of parasites being transferred to the human agent with each bite. We use a simple multiplicative model for the relative risk, *φ_lik_*, due to the *l*-th vector for the *i*-th parasite in cell *k*:
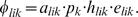
(1)Constraints on available data force further simplification of the model: (i) differences between the two primary parasites for malaria in Africa (*Plasmodium vivax* and *P. falciparum*) were ignored; (ii) the HBI was interpreted as an intrinsic property of the vector species that does not vary over geographical space; and (iii) differences in transmission efficiency between vectors were ignored. This results in the simplified relative risk model:
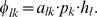
(2)This equation expresses the relative number of individuals in cell *k* to whom the malaria parasite is expected to be transmitted by the *l*-th vector. Finally, a linear correlation is assumed between the expected relative abundance of a vector and its relative probability of presence as predicted by its niche model. This assumption must be tested in the field. Because of these four assumptions, the relative risk model presented here must be regarded as very preliminary and treated with caution.

To construct a relative risk map from this model, the relative risk from the different vectors must be compounded for each cell in the landscape. Three different models for compounding relative risk were used for this purpose:

#### (i) Additive model

The relative risk due to the different vectors were added together and then normalized on a scale of 0 to 1. Let Φ*_u_* be the relative risk of cell *k*. Then:
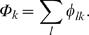
(3)This model thus assumes that there is no interaction between vector species, that each vector is able to inhabit the full extent of its fundamental niche and that there is no competition between vectors for human blood meals. If there is no such interaction then it should be possible to add the relative risks posed by the vectors in arriving at the overall relative risk posed to a given cell. However, the presumed lack of interaction between vectors has been questioned [36] and probably does not hold for all vector species. Moreover, given the methodology of this paper, there is an additional problem with the additive model. The relative probabilities provided by Maxent sum to 1 over the landscape. Thus, even if the relative abundances of a vector in the cells are linearly correlated with these probabilities, there is no way to estimate the absolute abundance. Adding the relative risk values makes the assumption that the highest absolute abundance of a vector among the cells of the landscape is the same for each vector. This assumption is ecologically suspect. Consequently, the next two models for compounding relative risk are more plausible than the additive model.

#### (ii) Minimax model

The relative risk of each cell was defined as the maximum relative risk from any one of the vectors. Thus:
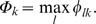
(4)This is being called a minimax model since, ultimately, in epidemiology, the goal is to minimize the risk of disease while what is being used as a relative risk measure is the maximum risk associated with all the vectors in a given cell.

#### (iii) Competitive exclusion model

The relative risk associated with the vector of highest relative abundance is identified with the relative risk of the cell. Let 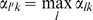. Then:
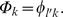
(5)Since the relative abundances were identified with relative probabilities of occurrence, this model assumes that the vector that has the highest predicted probability of occurrence will displace all others. This assumption is similar to that about a dominant vector incorporated in the risk maps of Kiszewski *et al*. [8]. However, as studies of malaria transmission have identified the presence of more than one significant malaria vector within some regions [37], [38] there is again reason to doubt the validity of this assumption in many contexts.

### Sensitivity Analysis

A sensitivity analysis was performed to determine the robustness of our results to variation in the values of the parameters included in the models. For each of the 100 niche models produced for each of the *Anopheles* species, 10 sets of HBI values were obtained by drawing randomly from a uniform distribution over the interval defined by the minimum and maximum measured HBI values for each species. For each of the three models, 1 000 relative risk values were produced for each cell using the 100 niche models, the random HBI values, and the actual human population densities. The standard deviation of the relative risk values of each cell was calculated and used to identify areas of high sensitivity.

## Results

**Figure 2:**
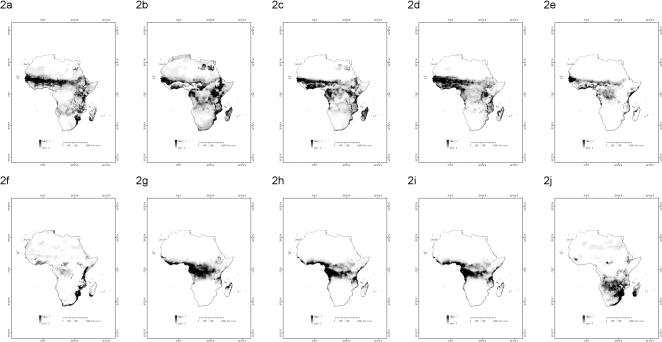
The distributions of 10 malaria vectors in Africa. Distributions are provided for: (a) *A*. *arabiensis*; (b) *A*. *coustani*; (c) *A*. *funestus*; (d) *A*. *gambiae*; (e) *A*. *melas*; (f) *A*. *merus*; (g) *A*. *moucheti*; (h) *A*. *nili*; (i) *A*. *paludis*; (j) *A*. *quadriannulatus*.

The need to set aside 25% of the records of each *Anopheles* species for testing purposes resulted in the production of niche models for only those 17 (out of 29) species for which four or more records were available. Data representing the accuracy of the niche models are provided in Table 3. Of these 17 niche models all but one possessed an AUC greater than 0.75. However, only 10 of the niche models possessed a *p* value less than 0.05 at a threshold of 0.10. As would be expected, the *p* values of the niche models were closely correlated with the number of records upon which the models were based. Niche models with an AUC greater than 0.75 and a *p* value less than 0.05 were produced for *A*. *arabiensis*, *A*. *coustani*, *A*. *funestus*, *A*. *gambiae*, *A*. *melas*, *A*. *merus*, *A*. *moucheti*, *A*. *nili*, and *A*. *quadriannulatus*. Figure 2 presents maps of the predicted geographical distributions of these species. Darker regions are those of greater relative probability of occurrence while lighter areas are those in which the relative probability of occurrence is small.

**Table 3:** Accuracy of the Niche Models

Species	AUC	Omission Rate	*p* Value
*A. arabiensis*	0.909	0.0722	<1.0E-6
*A. coustani*	0.952	0.000	6.22E-3
*A. funestus*	0.948	0.0529	<1.0E-6
*A. gambiae*	0.914	0.0782	<1.0E-6
*A. hancocki*	0.987	0.000	0.145
*A. marshallii*	0.856	0.000	0.226
*A. melas*	0.993	0.00715	<1.0E-6
*A. merus*	0.988	0.0114	<1.0E-6
*A. moucheti*	0.993	0.000	3.86E-3
*A. nili*	0.979	0.000	2.19E-3
*A. paludis*	0.977	0.000	0.0157
*A. pharoensis*	0.869	0.245	0.145
*A. pretoriensis*	0.656	0.000	0.508
*A. quadriannulatus*	0.941	0.154	<1.0E-6
*A. rufipes*	0.853	0.000	0.0924
*A. squamosus*	0.920	0.000	0.641
*A. ziemanni*	0.879	0.333	0.170

100 niche models were produced for each of the species listed in column (i). Column (ii) lists the average area under the curve of each model. Column (iii) lists the average omission rate of each model. Column (iv) lists the average *p* value of each model.

Two different tests were performed to determine the contributions of each of the environmental parameters to the niche models. In the first test, the AUC of each niche model was calculated using each of the environmental parameters individually. Those parameters that resulted in the highest AUC were interpreted as those which possessed the most information regarding the niche of a species. In the second test, the AUC of each niche model was calculated after omitting each of the environmental parameters one at a time. The effects of omitting each parameter were determined by comparing the resulting AUC to the actual AUC of the model. Those parameters for which the difference between these values was highest were interpreted as possessing the most information not present in the other environmental parameters.

The results of these two tests are provided in Table 4. As can be seen, there was no clear pattern in the contributions of the environmental parameters. The niches of different species appeared to be determined by different parameters. Over the entire data set of ten species, none of the parameters appeared to be significantly more important than any other parameter.

**Table 4:** Contributions of the Environmental Parameters

Species	Parameters Producing the Largest AUC When Included Separately			Parameters Producing the Smallest AUC when Omitted		
*A. arabiensis*	Temperature Seasonality, 0.772 (0.008)	Mean Temperature of Wettest Quarter, 0.760 (0.007)	Annual Precipitation, 0.746 (0.007)	Precipitation of Warmest Quarter, 0.890 (0.006)	Altitude, 0.893 (0.006)	Precipitation of Wettest Month, 0.894 (0.006)
*A. coustani*	Temperature Seasonality, 0.902 (0.007)	Temperature Annual Range, 0.825 (0.008)	Precipitation of Wettest Quarter, 0.796 (0.008)	Precipitation of Coldest Quarter, 0.892 (0.012)	Altitude, 0.905 (0.008)	Temperature Seasonality, 0.927 (0.008)
*A. funestus*	Precipitation of Wettest Month, 0.838 (0.003)	Temperature Seasonality, 0.831 (0.003)	Temperature Annual Range, 0.830 (0.003)	Precipitation of Wettest Month, 0.926 (0.004)	Minimum Temperature of Coldest Month, 0.935 (0.004)	Precipitation of Warmest Quarter, 0.938 (0.003)
*A. gambiae*	Mean Temperature of Coldest Quarter, 0.794 (0.005)	Minimum Temperature of Coldest Month, 0.780 (0.013)	Precipitation of Wettest Month, 0.838 (0.003)	Altitude, 0.891 (0.006)	Precipitation of Warmest Quarter, 0.898 (0.004)	Annual Precipitation, 0.901 (0.005)
*A. melas*	Altitude, 0.961 (0.020)	Mean Temperature of Wettest Quarter, 0.937 (0.020)	Precipitation of Wettest Month, 0.911 (0.007)	Precipitation of Coldest Quarter, 0.987 (0.005)	Precipitation of Warmest Quarter, 0.989 (0.006)	Landscape, 0.990 (0.003)
*A. merus*	Precipitation of Driest Month, 0.922 (0.012)	Precipitation of Coldest Quarter, 0.912 (0.029)	Altitude, 0.884 (0.012)	Precipitation of Warmest Quarter, 0.982 (0.005)	Altitude, 0.982 (0.004)	Mean Temperature of Driest Quarter, 0.984 (0.008)
*A. moucheti*	Temperature Annual Range, 0.980 (0.016)	Mean Diurnal Range, 0.965 (0.036)	Isothermality, 0.965 (0.036)	Landscape, 0.985 (0.003)	Precipitation of Coldest Quarter, 0.990 (0.005)	Mean Temperature of Driest Quarter, 0.991 (0.009)
*A. nili*	Temperature Annual Range, 0.982 (0.016)	Mean Diurnal Range, 0.966 (0.036)	Isothermality, 0.966 (0.036)	Mean Temperature of Wettest Quarter, 0.9623 (0.004)	Precipitation of Coldest Quarter, 0.968 (0.004)	Min Temperature of Coldest Month, 0.973 (0.003)
*A. paludis*	Temperature Annual Range, 0.984 (0.017)	Mean Diurnal Range, 0.968 (0.036)	Isothermality, 0.968 (0.036)	Temperature Annual Range, 0.970 (0.004)	Precipitation of Coldest Quarter, 0.974 (0.004)	Precipitation of Driest Quarter 0.976 (0.004)
*A. quadriannulatus*	Precipitation of Warmest Quarter, 0.874 (0.003)	Precipitation of Wettest Quarter, 0.863 (0.002)	Mean Temperature of Driest Quarter, 0.856 (0.003)	Mean Temperature of Driest Quarter, 0.913 (0.007)	Precipitation of Warmest Quarter, 0.932 (0.006)	Mean Temperature of Coldest Quarter, 0.934 (0.007)

Column (i) lists the 10 *Anopheles* species for which niche models were constructed. Columns (ii–iv) list the three parameters that produced the largest AUC when taken individually. These parameters are listed in decreasing order from (ii) to (iv) on the basis of their associated AUC values. Thus column (ii) lists the environmental parameter that possesses the most information regarding the niche of each species. Columns (v–vii) list the three parameters that produced the smallest AUC when omitted. These parameters are listed in increasing order from (ii) to (iv) on the basis of their associated AUC values. Thus column (v) lists the environmental parameter that possesses the most information not possessed by the other parameters regarding the niche of each species. Average AUC values are provided next to each environmental parameter with the standard deviation of the values provided in parenthesis.

Data representing the HBI values of the *Anopheles* species are provided in Table 5. HBI values were available for 9 of the 10 species for which niche models were produced. An HBI value for *A*. *plaudis* could not be found in the literature. The HBI values of the remaining species were observed to vary significantly, with mean values ranging between 0.011 for *A*. *quadriannulatus* and 1.00 for *A*. *merus*. The values averaged to obtain the mean HBI value were likewise found to vary for different species, with standard deviations as high as 0.269 for *A*. *melas* and 0.241 for *A*. *arabiensis*.

**Table 5:** Human Blood Index Values

Species	Mean	Standard Deviation	References
*A. arabiensis*	0.526	0.241	[67], [71], [85], [101], [103], [108], [111], [181]–[194]
*A. coustani*	0.157	0.019	[52], [101]
*A. funestus*	0.844	0.191	[67], [111], [129], [146], [184], [185], [191], [195]–[199]
*A. gambiae*	0.815	0.159	[54], [85], [103], [108], [129], [155], [181], [184], [185], [188]–[193], [200]
*A. melas*	0.576	0.269	[54], [103], [181]
*A. merus*	1.00	-	[191]
*A. moucheti*	0.931	0.080	[103], [129], [184]
*A. nili*	0.949	0.055	[52], [129], [184]
*A. paludis*	-	-	-
*A. quadriannulatus*	0.011	-	[183]

A list of the species is included in column (i). Columns (ii) and (iii) list the mean and standard deviation of the HBI values for each species. A list of the references from which the HBI values were drawn is provided in column (iv).

**Figure 3:**
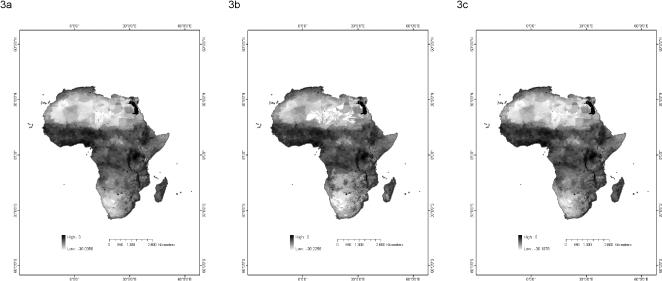
The distribution of malaria relative risk in Africa. Three different types of risk were calculated as follows: (a) the probability of occurrence of each vector in each cell was multiplied by both the human population density of the cell and the HBI of the vector. The relative risk of malaria in the cell was calculated as the sum of these values; (b) the vector possessing the maximum probability of occurrence was identified for each cell. Its probability of occurrence was multiplied by its HBI and the human population density of the cell. The relative risk of malaria in the cell was calculated as the product of these three values; and (c) the probability of occurrence of each vector in each cell was multiplied by the human population density of the cell and the HBI of the vector. The relative risk of malaria in the cell was calculated as the maximum of these values. The maps plot the natural logarithm of the relative risk.

Figure 3 depicts the relative risk maps constructed using the additive, minimax, and competitive exclusion models. Cells in each map were assigned a value between 0 and 1, with 0 representing no risk and 1 representing maximal relative risk. The resulting relative risk values were found to congregate closely to either 0 or 1. To ease the visual discernment of the relative risk faced in different regions, the maps in Figure 3 plot the natural logarithm of the relative risk values.

In general the relative risk maps produced by the three relative risk models were quite similar. As can be seen in Figure 3, the maps produced from the minimax and competitive exclusion models were nearly identical. These maps differed slightly from the relative risk map produced using the additive model as the relative risk in the additive model was more closely restricted to the areas of high population density. As can be seen in comparing Figure 1 with Figure 3, population density appears to have been the primary determinant of the relative risk of malaria in each of the three models. Areas of high human population density were found to be those in which the relative risk of malaria was greatest. The sole exception to this was in North Africa in which high human population densities were accompanied by relatively low relative risk values.

The sensitivity of the additive model was observed to be less than that of the competitive and minimax models. The standard deviations in the relative risk values ranged from 0 to 0.144 for the additive model to 0 to 0.280 for the competitive model and 0 to 0.279 for minimax model. In each model the majority of cells possessed a standard deviation less than 0.001. In the additive model 98.9% of cells had a standard deviation less than 0.001, while the percentages for the competitive and minimax models were 97.7% and 98.5%, respectively. Most cells were thus robust to variation in the parameter values. There was no clear pattern observed in the distribution of sensitivity across the landscape. While relative risk was concentrated in areas of high human population density, sensitivity was not.

## Discussion

As explained below, our relative risk maps are preliminary and must be treated with due caution. However, the methodology developed here can be used to construct relative risk maps for other infectious diseases, with the risk models modified to reflect the presence of multiple agents and reservoirs, more complicated modes of transmission, and other relevant ecological factors. Because, as we and others have shown [9], [11], [16], [17], niche models of acceptable accuracy based on climatic and topographic parameters (as determined by the internal tests of software packages as well as from comparisons of maps produced by experts—see below for more discussion) can be constructed with sparse data, this methodology shows promise for the construction of relative risk maps for infectious diseases even when data are limited and not easily collected. Some suggestive recent work argues that, besides climatic and topographic factors, land use and land cover change influence the spread of malaria [39], [40]. The models presented here used land cover as one of the explanatory variables in the niche models; in future work we plan to include land use and to explore the effects of land cover in more detail, using a finer classification of land cover types.

A variety of different climatic and topographical factors were important for predicting the distributions of different species. Levine *et al*. [23] report similar variable results for the three *Anopheles* species that they model. These results have the implication that, to predict malaria vector species distributions, simple ecological heuristic rules (for instance, those incorporating optimal precipitation and/or temperature) will likely be unreliable and should be replaced with predictions of niche models based on as complete a set of environmental parameters as possible. Rogers and Randolph [41] have also noted the lack of relatively simple heuristic rules.

A perhaps not unexpected result is that the most important determinant of relative risk for malaria was human population density, assuming as we do here for Africa that (i) ecological and demographic factors rather than control measures are the determinants of risk and (ii) parasite densities are sufficiently high for disease spread. The extent of the effect of population density in this analysis is likely a result of the multiplicative nature of our risk models. However, this effect cannot be regarded solely as an artifact of the admitted simplicity of these models as any plausible model of infectious disease transmission should include the human population as a multiplicative factor. This dependence on population density has the implication that the expected local increase of population density due to increased urbanization [42], especially in Africa [43], will increasingly exacerbate the risk of malaria unless control measures are implemented [39].

Turning to the details of our results, with a few notable exceptions, the distributions for the niche models in Figure 2 closely follow the previously proposed distributions of most modeled species. This can be seen by comparing by region the vector distributions provided in Figure 2 with those previously presented in the literature.

The niche models of vectors within West Africa appear to coincide quite closely with the expert-based distributions of the modeled vectors within this region. Haworth [34] identified *A*. *arabiensis*, *A*. *coustani*, *A*. *funestus*, *A*. *gambiae*, and *A*. *nili* as primary and secondary malaria vectors in West Africa. This is consistent with the niche models of Figure 2 in which these three species are shown to possess extremely high relative probabilities of occurrence within this region. Haworth [34] also identified *A*. *melas*, *A*. *moucheti*, and *A*. *paludis* as vectors with limited presence in West Africa. This is consistent with the models in Figure 2 in which these species have limited distributions in parts of that region. In addition, Gillies and Coetzee [44] presented this region as containing *A*. *arabiensis*, *A*. *gambiae*, and *A*. *melas*, while White [45] reported the region as containing *A*. *arabiensis*, *A*. *gambiae*, *A*. *melas*, and *A*. *funestus*. The distributions of these species within this region provided by these two sources were thus consistent with the niche models of Figure 2.

In Southeast Africa and Madagascar the niche models again correspond closely to expert-based distributions. Haworth [34] identified *A*. *arabiensis*, *A*. *coustani, A*. *funestus*, *A*. *gambiae*, *A*. *merus*, and *A*. *paludis* as the primary and secondary malaria vectors within the region. In Figure 2 these species have high relative probabilities of occurrence in the region. Moreover, the distributions within this region of *A*. *arabiensis*, *A*. *gambiae*, and *A*. *merus*, as presented by Gillies and Coetzee [44], and the distributions of *A*. *arabiensis*, *A*. *gambiae*, *A. funestus*, and *A*. *merus*, as presented by White [45], were consistent with the niche models in Figure 2.

While the correspondence between the niche models produced in this analysis and the expert-based distributions of the *Anopheles* species was quite strong in most regions, discrepancies between these distributions are found in Central Africa. Gillies and Coetzee [44] presented the distributions of *A*. *arabiensis* and *A*. *gambiae* as stretching across Central Africa. However, these distributions conflict with the niche models of Figure 2, in which both vectors have low probabilities of occurrence throughout this region. In slight contrast to Gillies and Coetzee, White [45] depicted both *A*. *gambiae* and *A*. *funestus* as present in Central Africa, while depicting *A*. *arabiensis* as largely absent from the region. While this distribution of *A*. *arabiensis* agrees with the niche models of Figure 2, the proposed distributions of *A*. *gambiae* and *A. funestus* do not. Levine *et al*. [23] found both *A*. *arabiensis* and *A*. *gambiae* to be distributed throughout Central Africa, unlike what is seen in Figure 2. Similar results were obtained by Lindsay *et al.* [20] who found the climate throughout Central Africa to be suitable for both of these species. Rogers *et al.* [22] obtained results similar to those of both Levine *et al*. and Lindsay *et al*. with respect to the distribution of *A*. *gambiae*, yet found *A*. *arabiensis* to be largely absent from Central Africa (which thus agrees with Figure 2).

These discrepancies may simply be a result of the general lack of distributional data for *Anopheles* species in Central Africa. Fewer than 10 occurrence records within this region were available for use in the present analysis, with the other analyses likewise lacking much data for the region. If the niche models produced by Maxent are excessively conservative, then the low probability of occurrence associated with *A*. *gambiae* in Central Africa may be a consequence of the lack of any occurrence data for the species in this region. However, given the current absence of records of *Anopheles* species within Central Africa, the attribution of a low relative probability of occurrence to both *A*. *arabiensis* and *A*. *gambiae* within the region may be a correct prediction. The high AUC values associated with the niche models produced in this analysis support this claim. Field data from Central Africa are required to resolve the discrepancies between our predictions and other distributional maps..

Some other discrepancies appear to indicate that our models are an improvement over previous proposed distributions. For example, Coetzee [46] criticized the niche models of Levine *et al*. [23] for predicting the occurrence of *A*. *quadriannulatus* in areas of central Botswana that are supposed to be too arid for the species. She also questioned proposed distributions of Lindsay *et al*. [20] for including *A*. *arabiensis* in arid parts of South Africa. In contrast with these distributions, the niche models in Figure 2 predict small relative probabilities of occurrence for *A*. *quadriannulatus* and *A*. *arabiensis* in central Botswana and South Africa, respectively. While our results thus avoid these criticisms, since Levine *et al*.'s criticisms were meant to illustrate the general shortcomings of computer modeling, our results also argue against excessive skepticism about computational approaches in epidemiology.

Finally, the possibly questionable assumptions underlying our preliminary relative risk maps deserve explicit emphasis. The most important of these is that the relative abundances of species can be estimated using the relative probabilities of occurrence predicted by niche models. The relative risk maps based on the minimax model and, especially, the competitive exclusion model are less affected by this assumption than that based on the additive model as, in the former models, the relative probabilities of occurrence of the various vectors species were not aggregated. However, before the predictions of any of these models are fully accepted, the relationship between the predicted relative probabilities of occurrence of these species and their actual distributions must be empirically tested. The importance of such a test extends beyond epidemiology and will be relevant to all disciplines that use niche models (including conservation biology). It is essential to test this assumption for a wide variety of vector species before recommending its adoption. If the assumption holds, then absolute abundances, drawn from a portion of the area under investigation, can be used to calibrate the relative abundance predictions of the niche models, thus allowing for the determination of absolute abundances across the landscape for use in the risk models.

If the assumption does not hold, the situation is more difficult. In almost all epidemiological contexts, at the landscape or at larger scales, it is unlikely that the absolute abundances of vector species can be empirically measured for the entirety of the region of concern. It may be possible to obtain some measurements, and use traditional interpolation techniques such as splining and kriging to acquire estimates for other regions within the geographical boundaries of the measured cells. These can then be used along with the niche models to extrapolate across the landscape using statistical techniques such as regression to other areas that are predicted to be suitable (by the niche models) for the vector species. However, the accuracy of these traditional interpolation techniques in epidemiological contexts must likewise be empirically tested.

In addition to this primary assumption a number of secondary assumptions were made regarding the relationships between the vectors considered in this analysis. One such assumption was that the variable risk posed by *P*. *vivax* and *P*. *falciparum* could be ignored. As these two parasites differ both in the risk that they pose to humans [47] and in their geographical distribution [48], this assumption should be questioned. In this analysis the assumption was mandated by a lack of data on the varying abilities of most vectors to transmit these two parasites. A more complete analysis will require the explicit incorporation of the differential risk posed by these two parasites.

It was also assumed that the efficiency with which malaria parasites were transmitted, both to and from humans, is the same both for different vectors and in different regions. Since transmission efficiency varies between both species [49], [50] and regions [51], it should be explicitly included for a credible assessment of malaria risk. It was not included in this analysis due to a lack of data.

A similar caveat is needed with respect to the HBI values. Though each HBI value was treated as a species-specific parameter, these values are environmentally influenced. Measured HBI values for a species obtained at one region vary substantially from those measured at other regions, with the variation often depending primarily on the availability of human blood meals [52]. In addition, the data used to derive the HBI values used here were not obtained by random sampling; thus, there is reason to question whether these HBI values adequately reflect the varying anthrophilicity of the vectors. The spatial heterogeneity of HBI values also questions the use of a single HBI value for a vector. However, our sensitivity analysis shows that including such spatial heterogeneity would likely have had little impact upon the results. Relative risk would still have been shown to be concentrated in areas of high population density. Nevertheless, a more precise representation of malaria risk should consider such heterogeneity.

The questionable simplifying assumptions made in the construction of the preliminary relative risk maps presented in this paper show that to construct large-scale risk maps that go beyond the predicted relative probabilities of vector species presence will require much more data, and data of different types (including, for instance, abundance and transmission efficiency data) than what are now available in the literature. Furthermore, the use of more sophisticated transmission models than those on which Equations (1) and (2) are based will only require even more data. For disease risk analysis, research geared towards the acquisition of such data remain a high priority.
